# A Study on Phosphorous-Based Flame Retardants for Transparent PET Composites: Fire, Mechanical, and Optical Performance

**DOI:** 10.3390/polym17162191

**Published:** 2025-08-11

**Authors:** Sara Villanueva-Díez, Alberto Sánchez-de-Andrés

**Affiliations:** TECNALIA, Basque Research and Technology Alliance (BRTA), Parque Científico y Tecnológico de Gipuzkoa Mikeletegi Pasealekua, 2, E-20009 Donostia-San Sebastián, Gipuzkoa, Spain; sara.villanueva@tecnalia.com

**Keywords:** flame-retardant transparent PET, arylphosphates, DOPO, cyclotriphosphazene, phosphonated organic flame retardant, phosphorous-based flame retardant, printed electronics

## Abstract

Flame-retardant poly (ethylene terephthalate) composites (FR PET) have been developed with the potential to be used as substrates in applications where flexibility and transparency are required. Several phosphorous-based flame retardants of a different nature were selected here for compounding by melt blending with a low-molecular-weight PET polymer. The fire reaction, transparency, and mechanical properties were analyzed. TGA and cone calorimetry were used to elucidate the gas-phase and condensed-phase actions of flame retardants and their effectivity. Cone calorimeters showed an improved performance with the addition of flame retardants, particularly a reduction in generated heat, improving the FGI (fire growth index) value. However, a V0 classification (following the UL94 standard) was achieved only with the addition of an organic phosphonate, Aflammit PCO900, to the PET matrix. This behavior was linked to the early reaction of this flame retardant in the gas phase, in addition to a plastification effect that causes the removal of the polymer from the incident flame. The presence of flame retardants reduced the transparency of composites over the neat PET, but, nevertheless, a good optical performance remained. No special effect was observed on the crystallization parameters. Therefore, the increase in opacity can be attributed to the poor miscibility of flame retardants and/or differences in the diffraction index of the polymer and FR additives.

## 1. Introduction

Poly (ethylene terephthalate) (PET) is a lightweight, sustainable material that is recyclable and durable, with excellent mechanical, electrical, and optical properties. It is one of the polymers with potential for the development of flexible functional and housing electronic devices [1,2,3]. Specifically, flexible PET films/sheets have demonstrated great potential in several applications where transparency is desired, such as antennae that can be seamlessly embedded into surfaces such as vehicle windshields, architectural glass, solar panels, and digital displays without obstructing visibility or performance [4,5,6,7], and building-integrated photovoltaic (BIPV) systems [8,9].

Despite its huge potential, PET has some limitations due to its poor fire behavior. As an inherently flammable organic material, the combustion of PET produces a significant amount of heat and harmful gases [10], as well as dripping, which makes it difficult for this polymer to meet the requirements of many fire reaction standards. Improving the fire resistance of PET using sustainable flame retardants is a challenge that must be addressed in order to widen the application field of this polymer.

The mechanisms of action of flame retardants are commonly discussed in terms of (i) the gas-phase action by quenching oxygen and radicals, thus reducing the flame temperature [11]; and (ii) forming a barrier char layer that limits heat and mass flow in the condensed phase [12]. In PET, the random chain scission dominates thermal degradation, with the major product being acetaldehyde, formed at temperatures of up to 290 °C, along with smaller amounts of CO, CO_2_, and ethane and very small amounts of other fuels such as methane and benzene [13]. The complete absence of crosslinking reactions prevents potential char-forming reactions during PET combustion. Therefore, the most effective flame retardants for this polymer could be either the ones that promote flame inhibition in the vapor phase [14] or intumescent-based ones where the char promotion arises from the flame retardant itself [15].

The best example of gas-phase-reaction flame retardants is halogen-based ones (bromine), which have shown to be effective in improving the fire resistance of PET [16,17]. However, regulations tend to ban the use of these chemicals due to their negative health and environmental profile [18]. Alternatively, phosphorus additives are valid compounds used to improve the fire resistance of polymers, and these chemicals can act both in the gas and condensed phase [19].

To date, the main effort in improving the fire performance of polyesters has been carried out on textiles and not so much on polyester compounds to be used in other sectors, like the electric and electronic sectors. In the textile field, three pathways to endow PET fabrics with flame retardancy have been studied, copolymerization [20,21,22], blending [23], and finishing, where the flame-retardant effect comes from the intumescent performance of the covering external layer [4,24,25,26,27].

The incorporation of phosphorus-containing third monomers into macromolecular chains of PET via melt copolymerization is currently regarded as the most effective approach for producing flame-retardant polyesters [28,29,30,31,32,33,34,35]. This solution allows us to maintain the mechanical performance of the polymer at the same time as improving the reaction to fire [36]. Among the commercially available options, 2-carboxyethyl(phenyl)phosphinic acid (CEPPA) stands out as the most representative flame-retardant monomer [37,38].

Additive flame retardants that are incorporated into the matrix by melt blending [39] are relatively easy to implement industrially, so they are the first option for most commercial polymers and have been also studied in PET. Thus, the TAD flame retardant (DOPO derivative) has shown a good flame-retardant performance in PET, exerting a gaseous-phase radical quenching effect by releasing free phosphorus–oxygen radicals. This compound also promotes the formation of a carbon layer in the condensed phase, which reduces the release of flammable gases and inhibits combustion by isolating the feedback of the oxygen and combustion heat to the base material [40]. Other DOPO derivatives—DOPO-PEPA, AAM-DOPO, and the organic phosphonate, Aflammit PCO 960—have also shown good flame-retardant performance in PET, with different radical species activities identified in the gas phase [41]. FR systems based on aluminum phosphinates and modified POSS [42,43,44], Kaolin [45], or Sb_2_O_3_ [46] have shown flame-retardant efficiency based on self-charring and intumescence. A polymer composite based on the co-polymerization of PET, the cyclotriphosphazene derivative (PN6), and the methylsiloxane derivative (PPPMS) has shown good fire performance by the formation of an effective carbon layer and the simultaneous increase in the melt viscosity, reducing the inherent dripping character of PET [47]. HNCP, also a cyclotriphosphazene derivative, enhances flame retardance in PET (UL94 V0) at 5% of loading by improving the quantity of the formed char [48]. Another approach, specifically developed for textiles, is based on impregnation of the fabric with a flame-retardant solution, bis-phosphonomethyl-aminomethyl phosphonic acid (BPMAEPA) and 3-(trimethoxysilyl) propyl methacrylate (KH-570), which does not interfere with the PET combustion mechanism but protects the surface, delaying the polymer combustion [49].

The use of PET in other applications, such as electric and electronic devices, BIPVs, or screens, requires an improvement in its fire behavior, but other topics must be considered. One of the attributes that makes PET a suitable material for these applications is its high degree of transparency. Normally, the presence of additives, such as those needed to improve fire performance, can negatively affect this transparency. On one side, there is the effect of light scattering in non-homogeneous solid composites with materials with diffraction index values. On the other side, there is the influence of additives on the nucleation and crystallization phenomena inherent to PET [50,51], which could have a dramatic effect on the transparence of films/sheets, especially at wide wall thicknesses. Some studies have shown that this handicap limits their widespread use in material packaging and optoelectronic material fields [52].

Transparency is not one of the attributes normally required in textiles, so this topic has not been specifically considered in flame retardant development for PET to date. Consequently, further research is needed to find a flame retardant solution to balance fire resistance, mechanical performance, and transparency. This work intends to cover this gap not previously addressed in the field. In this study, several phosphorous-based flame retardants are used for melt blending with PET, and the resulting composites are systematically studied with special attention paid to thermal, mechanical, optical, and fire performance.

## 2. Materials and Methods

### 2.1. Materials

Poly (ethylene) terephthalate of low molecular weight, PET, SPRIT T0 (IV.-0.54) (from NOVAPET-SAMCA, Barbastro, Spain), is used as the polymer matrix. Different commercial phosphorous-based halogen-free flame retardants (FRs) were incorporated: Sacoflam 47201, DOPO-derivative (from Metadynea, Krems, Austria), PX-200, resorcinol bis (2.6-dixylenyl phosphate) (from Daihachi Chemical, Tokyo, Japan), Aflammit PCO900 flame retardant based on phosphonated organic compounds (from Thor, Barcelona, Spain), and HFR 693, hexaphenoxycyclotriphosphazene (from Green Chemicals, Desio, Italy).

### 2.2. Preparation of Samples

PET and flame retardants (except the liquid one, Sacoflam 47201) were dried overnight in an oven at 120 °C and 70 °C, respectively, before every sample preparation to prevent hydrolysis at the high temperatures applied during extrusion. Then, several formulations based on the PET matrix, containing different flame retardant rates but with the same percentage of phosphorus, 0.63%, were prepared by melt blending in a twin screw co-rotating extruder with an L:D of 25. The composition of each formulation and applied barrel temperatures (from the hopper to the die zone) and screw speeds are presented in Table 1. Those temperature profiles are defined according to the thermal stability of the flame retardants and the potential plastification effect that allows a reduction in the usual PET processing temperatures.

Polymer filaments from the nozzle were dipped in a cold-water bath, dried again at a temperature of 70 °C, and grinded in a cutting mill, SM 300 from Retsch, at 700 rpm and using a 6 mm mesh.

Test specimens of type 1A (1 mm thickness) for UV–visible characterization and 80 mm × 10 mm × 4 mm test specimens for flexion, impact, and UL94 characterization were prepared by an injection process in a Battenfeld 35 injection machine from the compounds previously dried over 12 h at 100 °C followed by 5 h at 140 °C. Test specimens were injection-molded under controlled conditions using a constant mold temperature of 60 °C.

The injection process involved a fixed injection time of 15 s and a variable injection volume ranging from 20 to 50 cm^3^/s, with a consistent pressure of 120 and 150 bar for the flexion and tensile specimens, respectively. Additionally, a variable temperature profile above 280 °C was employed during the molding process.

Finally, cone calorimetry (100 mm × 100 mm × 3 mm) evaluation test specimens were obtained by compression molding using an LP-S-50 (LabTech, Samutprakarn, Thailand) hydraulic press. Samples were preheated at 285 °C without pressure for 3 min (except for the Sacoflam 47201, where a temperature of 255 °C was utilized), and thereafter a first pressure cycle of 20 bar was applied for 2 min followed by a second pressure cycle of 40 bar for 1 min. A last cold cycle for 10 min was applied at a pressure of 40 bar, achieving a final temperature of 80 °C.

### 2.3. Thermal and Fire Behavior Characterization

The thermal stability of the samples was analyzed by thermogravimetric analysis (TGA) using a TGA Q550 thermal analyzer from TA Instruments. Samples were heated from room temperature to 900 °C at a heating rate of 10 °C/min under air atmosphere.

The cone calorimeter test is designed based on the principle of oxygen consumption to measure the combustion heat release of materials. It can simulate real fire scenarios and characterize the burning performance of materials, providing valuable references for material evaluation, design, and fire prevention. Typically, the radiation intensity of the test material is similar to a fire situation (25–75 kW/m^2^). In this study, tests were carried out under a heat flux of 35 kW/m^2^ for 900 s. The tested sample dimensions were 100 mm × 100 mm × 3 mm. The Time to Ignition (TTI, s), peak Heat Release Rate (HRRpeak, kW/m^2^), Time To reach the Heat Release Rate peak (ttHRRpeak, s), the Total Heat Release (THR, MJ/m^2^), Maximum Average Rate Heat Emission (MARHE, kW/m^2^), Total Smoke Production (TSP, m^2^), Smoke Extinction Area (SEA, m^2^/kg), and CO/CO_2_ ratio were recorded. Two repetitions of each sample were performed. The sample holder was covered with a metal mesh to prevent deformation and loss of material.

The flammability of the PET materials was also measured by the UL-94 vertical burning measurement using injected test specimens of 80 × 10 × 4 mm^3^.

Thermal transitions of the polymers were measured by duplicate by Differential Scanning Calorimetry (DSC) with a TA DSC Q100 equipped with an Intracooler. Ultra-pure nitrogen was used as the purge gas. Samples with 9.5 mg of approximate weight were encapsulated in aluminum pans. Indium standards were used as calibrates. Samples were heated from 25 °C to 280/300 °C at a heating rate of 20 °C/min; then, they were held at 280/300 °C to erase thermal history. Later, they were cooled to 25 °C at 20 °C/min. A second heating scan was recorded between 25 and 280/300 °C. The degree of crystallinity, $X_{c}$, was calculated as

(1) $$X_{c}=\frac{∆H_{m}}{∆H_{m}^{0}}\cdot 100$$

where $∆H_{m}\;$(J/g) is the experimentally obtained melting enthalpy of the sample and $∆H_{m}^{0}$ is the equilibrium melting enthalpy. A value of $∆H_{m}^{0}$ = 140 J/g reported by Netzsch [53] for neat PET was employed.

### 2.4. Optical and UV Shielding Performance Testing

The optical properties of the films were observed by a UV–visible spectrophotometer (Jasco V-670, Jasco Inc., Easton, MD, USA) in the 190–800 nm transmission mode. Before the test, all samples were cut into rectangular shapes (10 × 25 mm) from the injected 1A specimens (1 mm thickness).

### 2.5. Mechanical Characterization

Flexural mechanical tests were performed by using a universal testing machine according to ISO 178 [54]. The deformation speed was 2 mm/min, and the support separation was 64 mm.

Impact properties were tested following ISO 179 [55] test using V-notch rectangular test specimens.

## 3. Results and Discussion

### 3.1. Fire Behavior: TGA, Cone Calorimeter, and UL94

**Figure 1:**
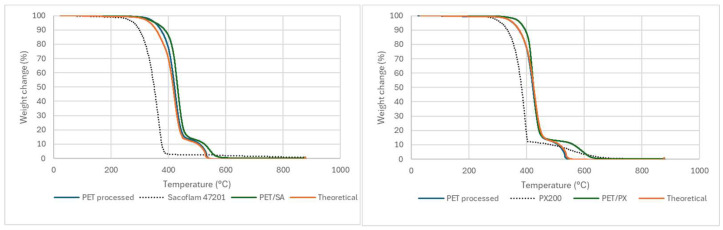

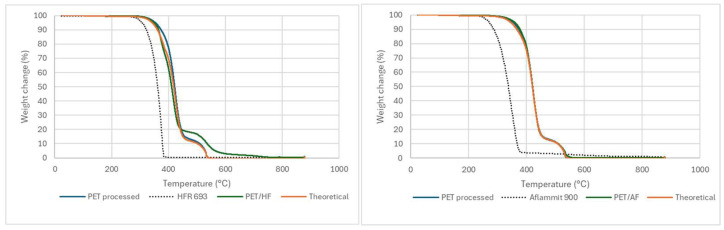
TGA of the selected formulations.

TGA thermograms shown in Figure 1 present the mass change of the samples (neat PET, flame retardants, and FR PET composites) as a function of temperature. All the flame retardants have shown good thermal stability beyond the processing temperature of PET, which is a requisite for its potential use in this polymer. For comparison purposes, each graphic shows the weight loss vs. temperature of neat PET, the corresponding flame retardant, and theoretical and real curves of the flame-retardant PET composites. The theoretical curves were calculated considering the ratio of the flame retardant and PET matrix in the composite and its respective contribution to the weight loss, assuming that no interaction occurs between them. Moreover, the onset degradation temperature (Ti) was considered to be at a loss of 5% of the initial mass, and the maximum mass loss rate temperature (T_max_) was determined by the maximum value of the first derivative. The mass that remained at 450 °C, 550 °C, and 700 °C, defined as residue, is shown in Table 2.

Neat PET thermally degrades in two steps, starting around 350 °C, without leaving residue over 550 °C. The first decrease in weight was assigned to the pyrolytic decomposition of PET, followed by a second reoxidation step at temperatures over 500 °C. The incorporation of flame retardants significantly affects the thermal degradation of the PET/FR blend. This is evident in the curves of PX200 and HFR 693, where the interaction of PET with the flame retardant gives a higher thermal stability to the generated char, a with decomposition temperature 50 °C higher than of the plain PET and with a residue that remained at 550 °C (Table 2). Furthermore, in the case of HFR 693, in addition to increasing the PET’s compound thermal stability, it also promotes the generation of additional char (see Table 2, residue at 450 °C).

It is well known that the incorporation of flame retardants modifies the degradation path of the polymer. Phosphorous-based flame retardants, active in the gas phase, release PO· radicals that quench the fire [56,57,58], but some of these compounds have shown certain activity in the condensed phase and can boost crosslinking/branching reactions in several polymers [59,60], including PET [61]. Thus, this action in the condensed phase was also reported for other resorcinol-based [47] and cyclotriphosphazene derivatives, which promotes the generation of highly phosphorous–carbon structured char in the condensed phase [62].

Aflammit apparently acts exclusively in the gas phase. This flame retardant decomposes through the cleavage of C–H and C-P bonds, mechanisms described recently [63].

**Figure 2:**
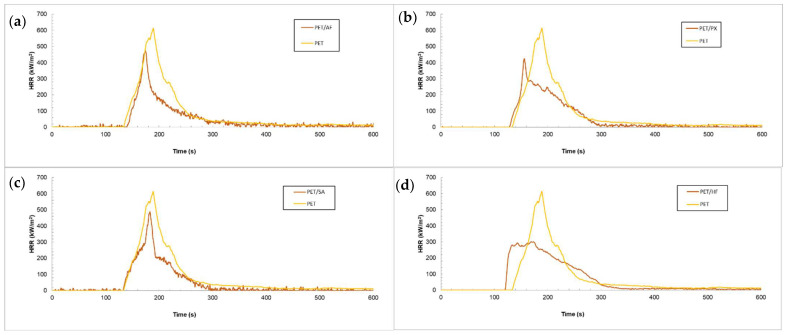
HRR vs. time plots comparing PET curve with FR composites: (**a**) PET/SA, (**b**) PET/PX, (**c**) PET/AF, and (**d**) PET/HF.

FR PET composites were tested in a cone calorimeter, and the results are shown in Table 3 and Figure 2. In this table, parameters related to heat release (HRR, THR, and MAHRE) and smoke generation (CO/CO_2_ ratio, SEA, and TSP) are shown, as well as with the Time to Ignition and Time to Heat Release Rate peak.

As shown in Figure 2, the peak Heat Release Rate (HRRpeak) of PET (yellow curve) was 569.7 kW/m^2^, while that of the flame-retardant PET materials decreased by 17.4% (PET/SA, blue curve, (a)), 22.6% (PET/PX, green curve, (b)), 17.7% (PETAF, grey curve, (c)), and 47.1% (PET/HF, brown curve (d)) compared to that of PET. The fire growth index (FGI) was defined as the ratio of PHRR and the time to PHRR (tPHRR), which represents the rate of fire spread during the burning process. The lower the value of the FGI, the higher the fire safety. The FGI value for the neat polymer, PET, was 3.2 kW/m^2^·s, reaching lower FGI values in the case of the composites, indicating an increased fire safety with the addition of flame retardants. A lower HRRpeak was attained with flame retardants acting both in the condensed and gas phase. Particularly, PET/HF showed an early ignition time and a reduction in heat emissions, with a curve corresponding to char-forming materials. Finally, the addition of flame retardants to the polymeric matrix increased toxicity-related parameters like the Total Smoke Production (TSP). Additionally, the non-complete combustion induced by flame retardants results in an increase in the CO/CO_2_ ratio [64].

**Figure 3:**
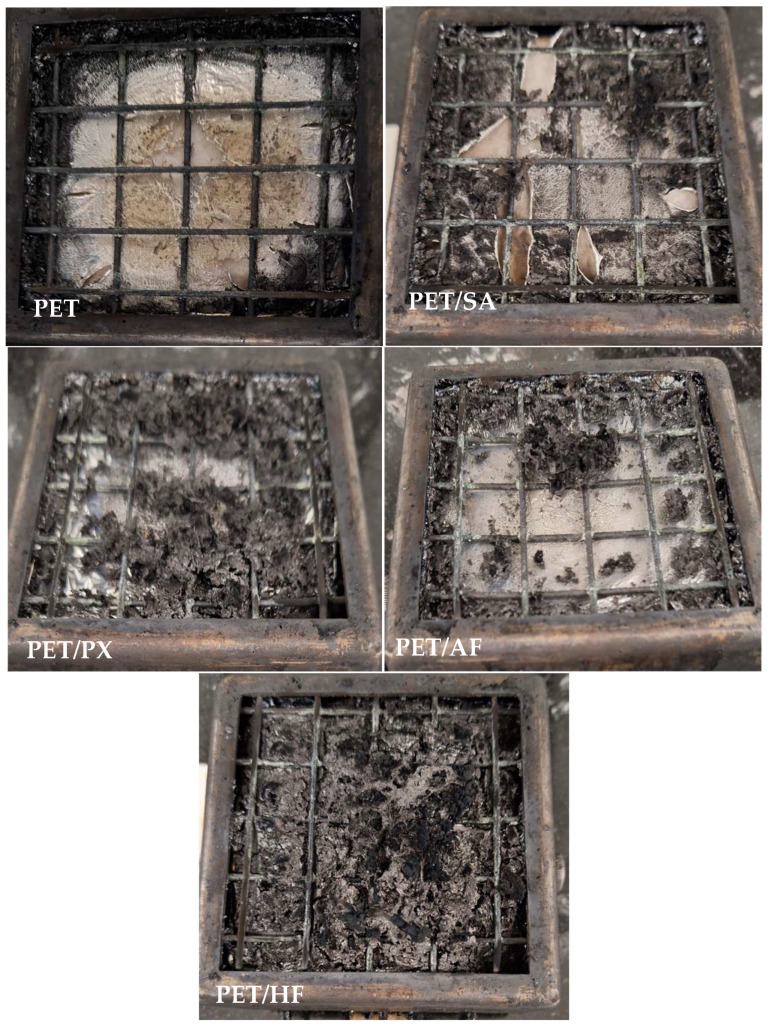
Obtained residues of the samples with different flame retardants studied by cone calorimeter.

Figure 3 shows pictures of the obtained residues for tested samples in the cone calorimeter. All materials present a residue, except for the neat PET matrix, with higher residual char in the PET/PX and PET/HF samples, which correlates with the previously shown TGA results.

Finally, the reference for the electric and electronic sector, the UL-94 vertical burning test, was conducted. The UL-94 test is regarded as a visual combustion characterization method. The corresponding values are presented in Table 4: ∑t_1_ represents the burning time (of five tested specimens) after being ignited for the first time, and ∑t_2_ (of the same five tested specimens) represents the burning time after being ignited for the second time.

As depicted in Table 4, neat PET exhibited visible flammability, burning within 117 s, and the cotton indicator was ignited by flaming drops. On the contrary, the PET formulation additivated with the Aflammit PCO 900 flame retardant (PET/AF) did not exhibit combustion, even though polymer dropping was still present (no flaming). The presence of the other flame retardants in the PET matrix decreased the after-flame time on the second ignition, as well as the total after-flame time. Meanwhile, in all these cases, the cotton ignited via flaming drops.

Flame retardant performance of traditional phosphorus-containing copolyesters is usually achieved by removing heat and fire from the material surface through melt dripping [65]. As an unwanted effect, the simultaneously produced droplets will lead to secondary burning and fire spread [66,67]. This is the performance observed in PET/SA, PET/PX, and PET/HF UL94 tests. The better result of Afflamit PCO 900 is attributed to two effects: firstly, the plasticizing effect that promotes the dropping of the PET polymer at lower temperatures, not enough to ignite the cotton, and, at the same time, the earlier reaction of this flame retardant (lower decomposition temperature), as shown previously in the TGA report. Both effects allow the prevention of ignition of the dropping polymer.

### 3.2. Effect of FR Addition on the Mechanical, Thermal, and Optical Properties of Composites

#### 3.2.1. Mechanical Properties

The mechanical properties of FR PET composites were analyzed following bending and impact standard tests, and the results are presented in Table 5.

The incorporation of the flame retardants into the PET matrix has a minor effect on the flexural and deformation strength parameters. The highest reduction in both parameters takes place with Sacoflam 47201. The flame retardant PX-200 shows a similar behavior to the PET matrix. The flexural modulus remains constant with the PX200 and HFR 693 flame retardants; however, it increases slightly with Sacoflam 4720 and Aflammit PCO 900.

The incorporation of the different flame retardants in the PET matrix has a negative effect on the impact properties, decreasing the impact resistance of the different PET/FR samples in comparison to the neat PET. However, this effect is more important in the case of Sacoflam 47201 and specially on Aflammit PCO 900. It is related to the higher plasticizing effect that these flame retardants have.

#### 3.2.2. Optical Properties

A known critical aspect in PET processing is the effect of cool crystallization on PET transparency. As an example, when extruding sheets above a certain thickness with no efficiency and fast cooling, the PET polymer chains have enough mobility to form crystals that appear in the inner areas of the sheet, which loses its transparency and becomes whitish. The addition of additives, such as flame retardants, can have a nucleating effect, leading to a higher crystallinity and transparence loss on FR PET composites.

The crystallization phenomena of the different composites were studied through Differential Scanning Calorimetry tests. In addition, the effects on transparency in injected parts with a thickness of 1 mm were verified by optical and spectroscopic methods.

- Crystallization of FR PET composites: DSC

DSC was utilized to analyze in duplicate the thermal-phase transitions of the PET samples. A first heating was carried out at a speed of 10 °C/min; in this stage, the material was melted, eliminating the thermal history that the material presents. Then, a cooling stage of 20 °C/min was carried out, where the exothermic crystallization peak was observed, and finally, a second heating curve again at 10 °C/min was run, where the endothermic melting peak was observed. Table 6 shows the main parameters measured.

**Figure 4:**
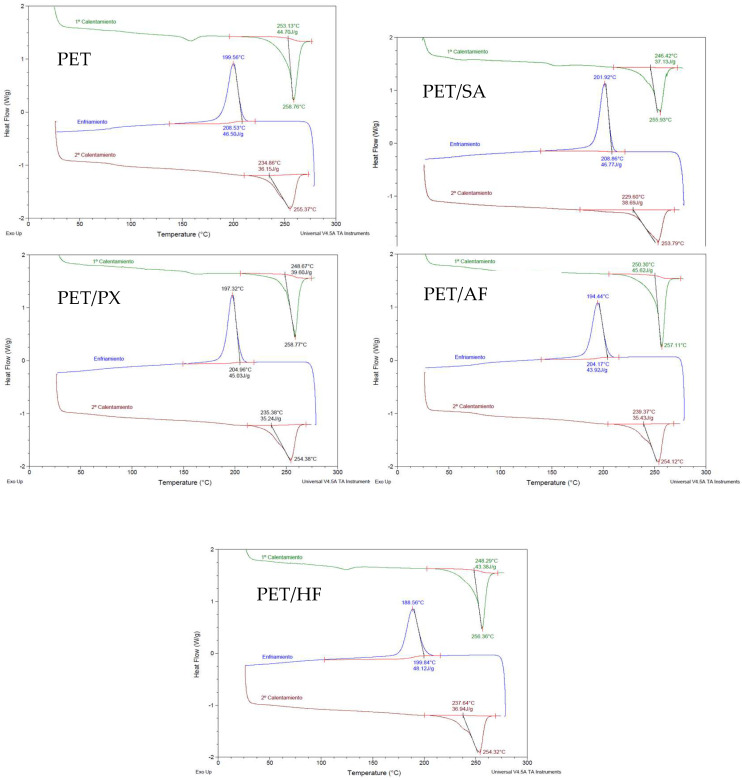
DSC curves of PET and flame-retardant composites.

The DSC curves of the different materials developed are presented in Figure 4.

The presence of flame retardants has a minor effect on melting and crystallization phenomena. As shown on Table 6, the melt onset temperature, Tm, of the FR PET composites is slightly lower than the neat PET sample. Moreover, the melting enthalpy (ΔHm), the percentage of crystallinity (Xc), and the crystallization temperature (Tc) of FR PET do not show remarkable differences with respect to neat PET.

The plasticizing effect of flame retardants is observed in the glass transition temperature region (Tg). This transition, clearly appreciated in the DSC of neat PET (77–88 °C), is not clear in the rest of modified FR PET, except for Aflammit, which shows a lower Tg (71–87 °C). This effect may also influence the observed decrease in the maximum crystallization temperature in most cases and the minimum reduction in melt temperature mentioned above. Alkyl/aryl phosphates of low molecular weight, of similar nature to some of the flame retardants studied in the present work, are among the earliest-known PVC plasticizers [68], and it is known that these types of plasticizers are incorporated in the amorphous parts of polymers, while the structure and size of any crystalline part remains unaffected [69]. This seems to be the effect of the flame retardants studied in the current work.

Previous studies have shown different performance with phosphorous-based FR reactive solutions that introduce phosphorous compounds on the molecular structure of PET [21], with a serious effect on the crystallization process and glass transition temperature. The presence of monomers like 2-carboxyethyl(phenyl)phosphinic acid (CEPPA) disrupts the chain regularity of PET, leading to a reduced crystallinity and a lowered melting temperature compared to neat PET. However, with this approach, the glass transition temperature increases, contrary to what was observed in the present work.

The presence of inorganic-based additive flame retardants as polyhedral oligomeric silsesquioxane (POSS) derivatives enhances the crystallinity of PET [43] by an effect of nucleating action. It is supposed that this increase in crystallinity could have a negative effect on transparence, an effect not analyzed in the referred study.

- Transparency: light transmission

**Figure 5:**
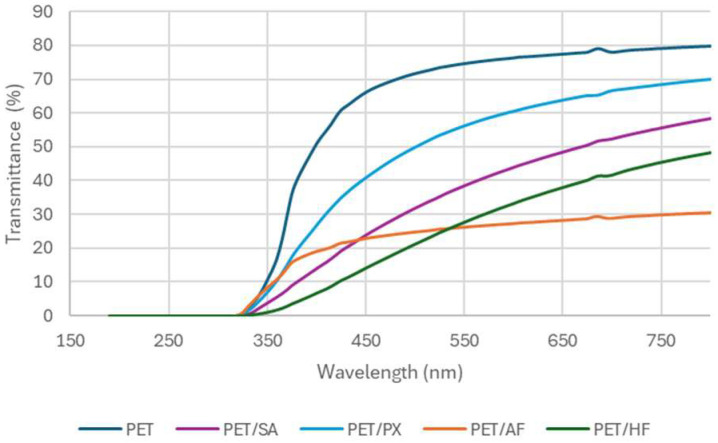
UV and visible transmittance (%) of the PET/FR formulations.

Figure 5 presents the transmittance spectra in the UV–VIS spectra of the injected test specimens of 1 mm thickness obtained from the different developed materials. The cooling time in the injection mold for specimens with thicknesses as low as 1 mm is supposed to be short enough to minimize crystallinity and therefore not interfere with the transparency of the film. Table 7 shows the transmittance values recorded at 560 nm.

**Figure 6:**
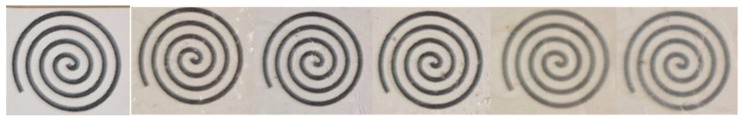
Optical analysis. From left to right: spiral curve and spiral curve covered by PET, PET/SA, PET/PX, PET/AF, and PET/HF.

Figure 6 shows the visual appearance and transparence of 1 mm thick films covering a spiral curve. Taking into account that neat PET is the material with better transparency, all the prepared FR/PET composites show relatively good performance, with no appreciable difference in the case of PET/SA, minor differences in the case of PET/PX, and a higher loss of transparency in the case of AF and HF flame retardants, probably owing to a worse miscibility between the flame retardant and the matrix.

## 4. Conclusions

In this study, fire performance and the mechanical and optical properties of PET composites containing different flame-retardant additives were investigated. The usual main mode of action of flame retardants was observed in the gas phase, but reaction on the condensed phase is also proposed for the PET/HF and PET/PX composites. Cone calorimeter testing showed that the FGI (fire growth index) value improves with the flame-retardant addition, especially with the FR acting in both phases, with the best results obtained in the case of PET/HF composites. However, a V0 classification (following the UL94 standard) was achieved only with the addition of 2.6 wt.% of Aflammit PCO900 to the PET matrix. This result is correlated with the early reaction of this additive in the gas phase and a plasticization effect that promotes PET dripping with no flaming drops.

Moreover, the transparency of the composites including flame retardants was reduced compared to the neat PET, but good optical performance was still observed. No special effect was noticed regarding crystallization, so this reduction in transparency may be related to differences in the refraction index of the polymer and the mentioned FR additives and to lower miscibility between the additive and the matrix. Despite this, the transparency was generally good in the low-thickness FR PET films, which makes these flame retardants a good option for PET protection in applications where good optical performance is required, such as printed electronics, screens, or BIPVs.

## Figures and Tables

**Table 1:** FR PET composition and melt extrusion conditions.

References	Composition PET/FR	T Profile (°C)	Screw Revolution (r.p.m.)
PET	PET 100	260/285/285/285/285	30
PET/SA	PET 93/Sacoflam 7	235/245/245/245/245	30
PET/PX	PET 92.73/PX 7.27	260/285/285/285/285	30
PET/AF	PET 97.4/Aflammit 2.6	260/270/270/270/270	30
PET/HF	PET 89.9/HFR 10.1	260/277/277/277/277	30

**Table 2:** T_i_, T_max_, and residual mass values obtained from TGA and DTGA thermograms.

Sample	T_i_ (°C)	T_max_ (°C)	Res_450_ (%)	Res_550_ (%)	Res_700_ (%)
PET	353 ± 2	423 ± 3	17.0 ± 0.1	0.0 ± 0.0	0.0 ± 0.0
PET/SA	348 ± 2	419 ± 2	17.1 ± 0.2	2.7 ± 0.1	0.2 ± 0.1
PET/PX	374 ± 4	424 ± 4	16.8 ± 0.1	11.3 ± 0.2	0.3 ± 0.1
PET/AF	357 ± 2	421 ± 3	16.5 ± 0.1	0.3 ± 0.0	0.0 ± 0.0
PET/HF	350 ± 3	372 ± 3	19.4 ± 0.2	7.0 ± 0.2	1.5 ± 0.1

**Table 3:** Cone parameters related to heat release and smoke generation of PET and FR PET.

Samples	Tignition (s)	HRRpeak (kW/m^2^)	tHRRpeak (s)	FGI (kW/m^2^·s)	THR (MJ/m^2^)	MARHE (kW/m^2^)	SEA (m^2^/kg)	CO/CO_2_ Ratio	TSP (m^2^)
PET	139.5 ± 2	569.7 ± 15	179.0 ± 10	3.2	41.0 ± 1.9	131.4 ± 4.4	711 ± 4	6.6 × 10^−3^	9.0 ± 1
PET/SA	151.0 ± 7	470.7 ± 18	184.0 ± 1	2.6	25.8 ± 0.7	87.7 ± 5.5	996 ± 10	2.7 × 10^−2^	13.0 ± 0.6
PET/PX	140.0 ± 0	441.0 ± 17	154.5 ± 2	2.9	35.5 ± 6.3	95.9 ± 2.7	871 ± 6	2.4 × 10^−2^	9.3 ± 2
PET/AF	157.0 ± 3	469.1 ± 3	176.5 ± 3	2.7	33.1 ± 5.7	91.3 ± 11.3	869 ± 15	2.3 × 10^−2^	12.2 ± 2
PET/HF	129.5 ± 1	301.4 ± 1	159 ± 10	1.9	44.2 ± 3.8	118.8 ± 0.5	713 ± 13	2.9 × 10^−2^	13.1 ± 0.1

**Table 4:** Detailed data of UL94 vertical burning test results.

Samples	∑t_1_ (s)	∑t_2_ (s)	∑ (t_1_ + t_2_) (s)	Rating	Comments
PET	37	80	117	V2	Cotton ignited by drops
PET/SA	18	8	26	V2	Cotton ignited by drops
PET/PX	22	14	36	V2	Cotton ignited by drops
PET/AF	0	0	0	V0	No cotton ignition
PET/HF	44	35	79	V2	Cotton ignited by drops

**Table 5:** Flexural and impact testing of plain and flame-retarded PET.

Sample	Flexural Modulus (MPa)	Deformation Strength (%)	Flexural Strength (MPa)	Charpy Impact Resistance (kJ/m^2^)
PET	1693 ± 128	3.6 ± 0.2	45.4 ± 1.4	2 ± 0.7
PET/SA	1915 ± 82	1.2 ± 0.7	22.6 ± 13.6	1.1 ± 0.2
PET/PX	1606 ± 29	3.6 ± 0.1	44 ± 0.7	1.6 ± 0.3
PET/AF	1733 ± 71	2.5 ± 0.2	40.5 ± 0.8	0.8 ± 0.1
PET/HF	1615 ± 132	3.3 ± 0.1	39.8 ± 0.5	1.7 ± 0.3

**Table 6:** Detailed phase-change properties of PET samples.

Samples	Melting Process		Crystallization Process		Tg (°C)	X_c_ (%)
	Tm (°C)	∆Hm (J·g^−1^)	Tc (°C)	∆Hc (J·g^−1^)		
PET	255 ± 1	38.6 ± 3.5	200 ± 2	45.0 ± 2.1	77–88	27.6 ± 2.5
PET/SA	253 ± 0	40.6 ± 2.7	202 ± 1	44.6 ± 3.1	--	29.0 ± 1.9
PET/PX	254 ± 0	36.9 ± 2.3	197 ± 2	43.9 ± 1.6	--	26.4 ± 1.7
PET/AF	253 ± 1	38.7 ± 4.6	184 ± 2	40.0 ± 5.6	71–87	27.7 ± 3.3
PET/HF	253 ± 1	37.8 ± 1.3	189 ± 1	41.0 ± 1.1	--	27.0 ± 0.2

**Table 7:** Transmittance values of the PET/FR samples.

Sample	Transmittance at 560 nm (%)
PET	75
PET/SA	40
PET/PX	57
PET/AF	26
PET/HF	29

## Data Availability

The data presented in this study are available on request from the corresponding author due to privacy reasons.

## Abbreviations

The following abbreviations are used in this manuscript:

PET	Polyethylene terephthalate
TGA	Thermogravimetric analysis
FGI	Fire growth index
FR	Flame retardant
DOPO	9,10-Dihydro-9-oxa-10-phosphaphenanthrene-10-oxide
TTI	Time to Ignition
HRRpeak	Heat Release Rate Peak
THR	Total Heat Release
MARHE	Maximum Average Rate Heat Emission
TSP	Total Smoke Production
SEA	Smoke Extinction Area
DSC	Differential Scanning Calorimetry
UV–VIS	Ultraviolet–Visible
